# Supplementary material for: The Impact of Purple-Flesh Potato (Solanum tuberosum L.) cv. “Shadow Queen” on Minor Health Complaints in Healthy Adults: A Randomized, Double-Blind, Placebo-Controlled Study
Source: Nutrients. 2022 Jun 13;14(12):2446. doi: 10.3390/nu14122446 (PMC9227939; doi:10.3390/nu14122446)
Supplement: Supplementary file 1 [file nutrients-14-02446-s001.zip › nutrients-1767048-supplementary.pdf]

Table S1. Key eligibility and exclusion criteria.

**Eligibility criteria:**
1. Subjects who have fully understood the significance, content, and purpose of this study and have given written consent to participate in this study.
2. Healthy Japanese men and women aged 50–70 years.
3. Subjects who have minor health complaints (assessed by a questionnaire for stress)
**Key exclusion criteria:**
1. Subjects who are under physician's advice, treatment, and/or medication for schizophrenia, depression, mania, neurological disorders, and/or sleep disorders.
2. Subjects with serious cerebrovascular, cardiac, hepatic, renal, gastrointestinal diseases, and/or affected with infectious diseases requiring reports to the authorities.
3. Subjects with a major surgical history relevant to the digestive system such as gastrectomy, gastrorrhaphy, and enterectomy.
4. Subjects with unusually high and/or low blood pressure and/or abnormal hematological data.
5. Subjects with severe anemia.
6. Pre- or postmenopausal women complaining of obvious physical changes.
7. Subjects who are at risk of having allergic reactions to drugs or foods especially based on potatoes, Betulaceae, and Poaceae.
8. Subjects who regularly take medicine, functional foods, and/or supplements that would affect sleep and/or stress.
9. Heavy smokers, alcohol addicts, or subjects with disordered lifestyle.
10. Subjects who donated either 400 mL whole blood within 16 weeks (women), 12 weeks (men), 200 mL whole blood within 4 weeks (men and women), or blood components within 2 weeks (men and women), before the current study.
11. Pregnant or lactating women or women who expect to be pregnant during this study.
12. Subjects who currently participate in other clinical trials, or participated within the last 4 weeks before the current study.
13. Any other medical and/or health reasons unfavorable to participation in the current study, as judged by the principal investigator.

Table S2. The change in hematological and biochemical parameters after taking "Shadow Queen" or placebo "Haruka" potato.

Biochemical parameter	Intervention	Week 0	Week 4	Week 8	ΔWeek 4	ΔWeek 8
Body fat ratio (%)	"Haruka"	30.21 ± 5.73	30.08 ± 6.37	30.19 ± 6.11	−0.14 ± 1.05	-0.02 ± 0.66
	"Shadow Queen"	25.21 ± 8.31	25.74 ± 9.11	26.11 ± 8.89 $^{\#}$	0.53 ± 1.16	0.90 ± 0.90
	p value	-	-	-	0.263	0.039 *
Body mass index (kg/m^2)	"Haruka"	22.45 ± 2.92	22.45 ± 2.94	22.50 ± 2.93	0.00 ± 0.46	0.05 ± 0.41
	"Shadow Queen"	20.79 ± 2.22	20.71 ± 2.09	20.93 ± 2.32	−0.07 ± 0.29	0.14 ± 0.28
	p value	-	-	-	0.730	0.624
Systolic blood pressure (mmHg)	"Haruka"	106.13 ± 14.34	109.25 ± 16.65	119.75 ± 17.81	3.13 ± 9.70	13.63 ± 13.63
	"Shadow Queen"	101.29 ± 8.98	99.0 ± 10.66	104.14 ± 10.27	−3.00 ± 7.56	2.86 ± 7.88
	p value	-	-	-	0.225	0.090
Diastolic blood pressure (mmHg)	"Haruka"	77.38 ± 10.42	78.50 ± 11.75	84.63 ± 10.01 $^{\#\#}$	1.13 ± 4.82	7.25 ± 4.80
	"Shadow Queen"	69.71 ± 6.82	71.50 ± 9.09	71.43 ± 9.80	1.67 ± 6.19	1.71 ± 9.09
	p value	-	-	-	0.857	0.156
Pulse (bpm)	"Haruka"	76.75 ± 8.07	77.88 ± 11.95	82.25 ± 21.59	1.13 ± 9.75	5.5 ± 17.16
	"Shadow Queen"	72.29 ± 11.27	72.17 ± 11.00	67.57 ± 9.03 $^{\#}$	−0.67 ± 8.57	−4.71 ± 3.99
	p value	-	-	-	0.727	0.149
Blood cell count						
WBC ($10^3/\mu L$)	"Haruka"	5.64 ± 2.08	4.95 ± 1.58	4.81 ± 1.52	−0.69 ± 1.42	−0.83 ± 1.50
	"Shadow Queen"	4.53 ± 1.04	4.50 ± 0.93	4.17 ± 0.79	−0.03 ± 0.41	−0.36 ± 0.61
	p value	-	-	-	0.258	0.456
RBC ($10^4/\mu L$)	"Haruka"	479.9 ± 40.0	468.1 ± 37.4	478.5 ± 41.2	−11.8 ± 15.0	−1.38 ± 14.1
	"Shadow Queen"	428.3 ± 45.9	437.1 ± 49.3	432.1 ± 41.9	8.86 ± 18.3	3.86 ± 17.0
	p value	-	-	-	0.032	0.526
Hg (g/dL)	"Haruka"	13.99 ± 1.13	13.76 ± 1.11	13.88 ± 0.98	−0.23 ± 0.41	−0.11 ± 0.34
	"Shadow Queen"	13.03 ± 1.05	13.34 ± 1.06	13.10 ± 0.99	0.31 ± 0.58	0.07 ± 0.55
	p value	-	-	-	0.055	0.446
Ht (%)	"Haruka"	42.89 ± 2.76	41.81 ± 2.89 #	42.11 ± 2.54	−1.08 ± 1.28	−0.78 ± 1.07
	"Shadow Queen"	40.09 ± 2.95	40.53 ± 3.10	39.51 ± 2.65	0.44 ± 1.65	−0.57 ± 1.51
	p value	-	-	-	0.067	0.766
Plt ($10^4/\mu L$)	"Haruka"	25.50 ± 3.52	25.51 ± 3.11	25.23 ± 3.73	0.01 ± 1.09	−0.28 ± 1.49
	"Shadow Queen"	24.19 ± 4.83	24.33 ± 4.60	23.14 ± 2.99	0.14 ± 1.45	−1.04 ± 3.75
	p value	-	-	-	0.846	0.602
Lipid parameter						
Total cholesterol (mg/dL)	"Haruka"	232.88 ± 33.22	230.25 ± 25.45	230.75 ± 31.08	−2.63 ± 16.44	−2.13 ± 14.68
	"Shadow Queen"	230.71 ± 30.76	239.43 ± 21.27	242.14 ± 16.79	8.71 ± 22.16	11.43 ± 19.85
	p value	-	-	-	0.277	0.153
HDL-C (mg/dL)	"Haruka"	78.00 ± 18.65	77.50 ± 18.34	77.50 ± 18.55	−0.50 ± 5.66	−0.50 ± 7.69
	"Shadow queen"	83.29 ± 22.37	84.14 ± 21.11	84.29 ± 18.71	0.86 ± 8.75	1.00 ± 7.83
	p value	-	-	-	0.723	0.715
LDL-C (mg/dL)	"Haruka"	136.88 ± 27.62	135.88 ± 26.37	131.38 ± 28.76	−1.00 ± 12.34	−5.50 ± 10.97
	"Shadow queen"	136.57 ± 18.07	139.86 ± 13.86	147.29 ± 12.62	3.29 ± 18.53	10.71 ± 14.41
	p value	-	-	-	0.602	0.028 *
Triglyceride (mg/dL)	"Haruka"	102.50 ± 96.11	123.38 ± 101.50	115.13 ± 81.27	20.88 ± 25.33	12.63 ± 17.25
	"Shadow queen"	52.43 ± 11.83	70.57 ± 31.33	80.43 ± 20.65 $^{\#}$	18.14 ± 26.65	28.00 ± 29.41
	p value	-	-	-	0.842	0.231
Glycometobolism assciated parameters						
Blood glucose (mg/dL)	"Haruka"	97.88 ± 28.74	96.38 ± 27.88	95.00 ± 24.69	−1.50 ± 5.93	−2.88 ± 7.81
	"Shadow Queen"	89.86 ± 5.61	87.29 ± 4.92	86.86 ± 5.46	−2.57 ± 6.48	−3.00 ± 6.88
	p value	-	-	-	0.743	0.974
HbA1c (%)	"Haruka"	5.54 ± 0.79	5.56 ± 0.74	5.78 ± 0.68 $^{\#}$	0.03 ± 0.07	0.24 ± 0.21
	"Shadow Queen"	5.20 ± 0.15	5.23 ± 0.19	5.49 ± 0.18 $^{\#\#}$	0.03 ± 0.08	0.29 ± 0.07
	p value	-	-	-	0.926	0.550
Liver function						
AST (U/L)	"Haruka"	19.88 ± 2.53	20.25 ± 3.28	19.63 ± 2.62	0.38 ± 2.33	−0.25 ± 1.28
	"Shadow Queen"	23.43 ± 3.26	22.00 ± 3.65 $^{\#}$	22.43 ± 3.60	−1.43 ± 1.27	−1.00 ± 2.38
	p value	-	-	-	0.092	0.452
ALT (U/L)	"Haruka"	17.13 ± 8.34	17.63 ± 8.02	16.88 ± 7.64	0.50 ± 2.93	−0.25 ± 1.49
	"Shadow Queen"	20.00 ± 7.53	18.43 ± 7.35	20.29 ± 9.53	−1.57 ± 3.64	0.29 ± 4.57
	p value	-	-	-	0.244	0.775
γ-GTP (U/L)	"Haruka"	17.13 ± 8.34	17.63 ± 8.02	16.88 ± 7.64	2.25 ± 4.89	1.88 ± 4.09
	"Shadow Queen"	20.00 ± 7.53	18.43 ± 7.35	9.53 ± 9.53	−1.43 ± 4.28	−0.43 ± 2.37
	p value	-	-	-	0.148	0.214

ALP (U/L)	"Haruka"	204.75 ± 47.22	204.13 ± 48.74	208.88 ± 46.68	−0.63 ± 9.68	4.13 ± 18.2
	"Shadow Queen"	216.00 ± 46.82	217.14 ± 45.37	219.43 ± 54.61	1.14 ± 19.52	3.43 ± 19.89
	p value	-	-	-	0.824	0.945
LDH (U/L)	"Haruka"	186.38 ± 21.10	188.00 ± 29.91	178.75 ± 21.16	1.63 ± 22.14	−7.63 ± 9.26
	"Shadow Queen"	190.43 ± 22.10	176.13 ± 19.39 ##	171.14 ± 17.75 ##	−14.29 ± 10.01	−19.29 ± 11.53
	p value	-	-	-	0.104	0.049 *
Renal function						
BUN (mg/dL)	"Haruka"	13.60 ± 2.96	13.23 ± 2.77	13.51 ± 2.71	−0.38 ± 1.51	−0.09 ± 2.46
	"Shadow Queen"	13.57 ± 2.52	14.74 ± 2.61	12.67 ± 1.56	1.17 ± 2.48	−0.90 ± 2.37
	p value	-	-	-	0.163	0.527
CRE (mg/dL)	"Haruka"	0.79 ± 0.20	0.78 ± 0.19	0.84 ± 0.20 ##	−0.01 ± 0.04	0.05 ± 0.03
	"Shadow Queen"	0.74 ± 0.11	0.77 ± 0.12	0.81 ± 0.10 #	0.03 ± 0.03	0.07 ± 0.04
	p value	-	-	-	0.086	0.208
Uric acid (mg/dL)	"Haruka"	5.21 ± 0.83	4.96 ± 1.04	4.98 ± 0.99	−0.25 ± 0.38	−0.24 ± 0.39
	"Shadow Queen"	4.84 ± 1.56	5.31 ± 1.33 #	5.06 ± 1.03	0.47 ± 0.34	0.21 ± 0.6
	p value	-	-	-	0.002 **	0.103
Diet survey						
Total dietary intake (kcal/day)	"Haruka"	2024.6 ± 427.6	-	2099.4 ± 391.2	-	74.8 ± 141.1
	"Shadow Queen"	1952.8 ± 407.5	-	1850.6 ± 322.79	-	−102.2 ± 208.8
	p value	-	-	-	-	0.073
Protein (g/day)	"Haruka"	74.2 ± 16.6	-	73.0 ± 15.9	-	−1.2 ± 4.8
	"Shadow Queen"	69.0 ± 10.3	-	64.9 ± 8.4	-	−4.1 ± 9.0
	p value	-	-	-	-	0.446
Fat (g/day)	"Haruka"	78.1 ± 23.6	-	80.5 ± 21.2	-	2.3 ± 7.6
	"Shadow Queen"	69.1 ± 16.9	-	62.3 ± 13.1 #	-	−6.8 ± 7.2
	p value	-	-	-	-	0.034 *
carbohydrate (g/day)	"Haruka"	249.6 ± 41.5	-	264.1 ± 41.7	-	14.4 ± 22.3
	"Shadow Queen"	255.1 ± 59.4	-	250.7 ± 50.4	-	−4.5 ± 38.7
	p value	-	-	-	-	0.260
Daietary fiber (g/day)	"Haruka"	14.1 ± 3.9	-	15.2 ± 3.2 #	-	1.1 ± 1.0
	"Shadow Queen"	14.3 ± 2.1	-	15.2 ± 3.3	-	0.9 ± 2.0
	p value	-	-	-	-	0.875
Salt (g/day)	"Haruka"	9.0 ± 2.4	-	9.9 ± 1.9 #	-	0.9 ± 0.9
	"Shadow Queen"	9.9 ± 2.1	-	10.1 ± 1.7	-	0.2 ± 1.3
	p value	-	-	-	-	0.214

Values are shown as the means ± standard deviation. **Δ,** changes; WBC, white blood cell; RBC, red blood cell; Hb, hemoglobin; Ht, hematocrit; Plt, platelet count; HDL-C, high density lipoprotein cholesterol; LDL-C, low density lipoprotein cholesterol; HbA1c, Hemoglobin A1c; AST, aspartate aminotransferase; ALT, alanine aminotransferase; γGTP, gamma-glutamic transaminase; ALP, alkaline phosphatase; LDH, lactate dehydrogenase; BUN, blood urea nitrogen; CRE, creatinine. Student's t-test for independent samples vs. placebo, paired t-test vs. 0 week was performed to analyze the values. Statistical significance, *; < 0.05, **; < 0.01 vs. placebo, #; < 0.05, ##; < 0.01 vs. 0 week.
